# Retrospective evaluation of vector-borne pathogens in cats living in Germany (2012–2020)

**DOI:** 10.1186/s13071-021-04628-2

**Published:** 2021-02-25

**Authors:** Ingo Schäfer, Barbara Kohn, Maria Volkmann, Elisabeth Müller

**Affiliations:** 1 LABOKLIN GmbH and Co. KG, Bad Kissingen, Germany; 2 Clinic for Small Animals, Faculty of Veterinary Medicine, Freie Universität Berlin, Berlin, Germany; 3 Institute of Veterinary Epidemiology and Biostatistics, Freie Universität Berlin, Berlin, Germany

**Keywords:** Arthropod-transmitted infections, Feline, Laboratory diagnostics

## Abstract

**Background:**

Blood-feeding arthropods can transmit parasitic, bacterial, or viral pathogens to domestic animals and wildlife. Vector-borne infections are gaining significance because of increasing travel and import of pets from abroad as well as the changing climate in Europe. The main objective of this study was to assess the percentage of cats with positive test results for selected vector-borne pathogens in Germany and explore any possible association of such results with time spent abroad.

**Methods:**

This retrospective study included test results from cats included in the “Feline Travel Profile” established by the LABOKLIN laboratory at the request of veterinarians in Germany between April 2012 and March 2020. This diagnostic panel includes the direct detection of *Hepatozoon* spp. and *Dirofilaria* spp. *via* PCR as well as indirect detection assays (IFAT) for *Ehrlichia* spp. and *Leishmania* spp. The panel was expanded to include an IFAT for *Rickettsia* spp. from July 2015 onwards.

**Results:**

A total of 624 cats were tested using the “Feline Travel Profile.” Serum for indirect detection assays was available for all 624 cats; EDTA samples for direct detection methods were available from 618 cats. Positive test results were as follows: *Ehrlichia* spp. IFAT 73 out of 624 (12%), *Leishmania* spp. IFAT 22 out of 624 (4%), *Hepatozoon* spp. PCR 53 out of 618 (9%), *Dirofilaria* spp. PCR 1 out of 618 cats (0.2%), and *Rickettsia* spp. IFAT 52 out of 467 cats (11%) tested from July 2015 onwards. Three cats had positive test results for more than one pathogen before 2015. After testing for *Rickettsia* spp. was included in 2015, 19 cats had positive test results for more than one pathogen (*Rickettsia* spp. were involved in 14 out of these 19 cats).

**Conclusions:**

At least one pathogen could be detected in 175 out of 624 cats (28%) *via* indirect and/or direct detection methods. Four percent had positive test results for more than one pathogen. These data emphasize the importance of considering the above-mentioned vector-borne infections as potential differential diagnoses in clinically symptomatic cats.

**Graphical Abstract:**

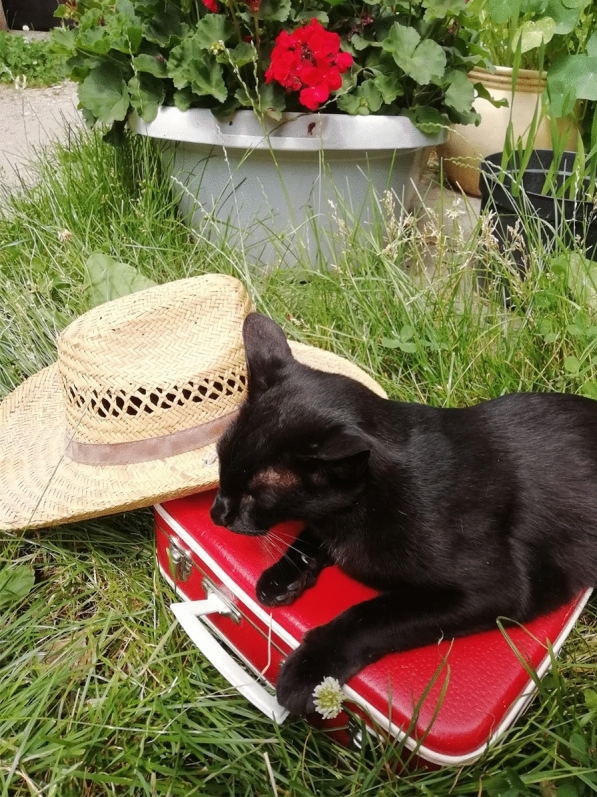

## Introduction

Cats are exposed to blood-feeding arthropods such as fleas, ticks, or mosquitoes, especially outdoor or stray cats without ectoparasite prophylaxis [1, 2]. These vectors can transmit parasitic, bacterial, or viral pathogens, which may subsequently cause infection in competent hosts such as cats. The occurrence of feline infectious agents is influenced mainly by the distribution of competent vectors, e.g. areas with high prevalences of *Leishmania* spp. were associated with habitats of phlebotomine sand flies in the Mediterranean and Southeast Europe [3]. *Hepatozoon* spp. are transmitted by various blood-feeding arthropods worldwide, including ticks, mites, sandflies, tsetse flies, lice, kissing bugs, and leeches [4, 5]. Infections with *H. felis* or, less frequently, *H. canis* and *H. silvestris* have been detected in cats in the Mediterranean and Southeast Europe [4–8]. There are also single case reports of infections with *H. felis* in Austria [9] and *H. silvestris* in Switzerland [8]. Infections with *Dirofilaria* spp., nematodes transmitted by mosquitoes, are less frequently reported in cats compared to dogs [2, 10]. While infections with *D. immitis* generally occur within the Mediterranean and Southeast Europe, there has been just one case report of a cat infected with *D. repens* in Poland [11]. *Rickettsia felis* has been detected in fleas in Germany [12], and as such autochthonous infections in cats in Germany are possible. Other documented vector-borne pathogens affecting cats in Europe include helminths (*Thelazia callipaeda*, *Dipylidium caninum*), bacteria (*Bartonella* spp., *Haemoplasma* spp., *Borrelia burgdorferi* complex, *Anaplasma (A.) phagocytophilum*, *A. platys*, *Coxiella burnetii*, *Francisella tularensis*), protozoa (*Babesia* spp., and *Cytauxzoon* spp.), as well as viruses, namely *Flaviviridae* [2].

Among the pathogens examined in this study, *Rickettsia* spp., *Leishmania* spp., and *Dirofilaria* spp. have zoonotic potential and are consequently of importance for public health in Europe [2]. To the knowledge of the authors, there are presently no studies regarding the prevalence of antigens and/or antibodies to the vector-borne pathogens *Leishmania* spp., *Ehrlichia* spp, *Rickettsia* spp., *Dirofilaria* spp., and *Hepatozoon* spp. in cats in Germany. The aims of this study were to determine the percentage of positive test results for these vector-borne pathogens in cats for which samples were provided by veterinarians in Germany to the veterinary laboratory (Bad Kissingen, Germany) and to determine whether positive results were associated with a background history of time spent abroad.

## Methods

This study included any “Feline Travel Profile” results of samples provided by veterinarians in Germany between April of 2012 and March of 2020. The panel includes a direct assay by polymerase chain reaction (PCR) of *Hepatozoon* spp. (TaqMan® real-time PCR, target: 18S rRNA) and *Dirofilaria* spp. ((TaqMan® real-time PCR, target: 5.8S rDNA, based on Rishniw et al. [13]). Furthermore, it includes immunofluorescence antibody testing (IFAT) for *Ehrlichia* spp. (MegaFLUO® EHRLICHIA canis, MegaCor Diagnostik GmbH, Hörbranz, Austria; ≥ 1:40 positive) and *Leishmania* spp. (MegaFLUO® LEISH, MegaCor Diagnostik GmbH, Hörbranz, Austria; > 1:64 positive) as well as *Rickettsia* spp. (RICKETTSIA CONORII IFA SLIDE, Viracell, Granada, Spain; > 1:128 positive) from July 2015 onwards (Table 1). Where possible, information on time spent abroad was collected in questionnaires and telephone calls to the treating veterinarians. A descriptive statistical analysis of the data collected was made using SPSS for Windows (version 27.0, SPSS Inc., Armonk, NY, USA).

**Table 1:** Results of the “Feline Travel Profile” diagnostic panel performed by the LABOKLIN laboratory (Bad Kissingen, Germany) in 624 cats from April 2012 until March 2020)

Time period	Total *n/N* (%)	Hepatozoon spp.^a,f^ *n/N* (%)	Dirofilaria spp.^b,f^ *n/N* (%)	Ehrlichia spp.^c^ *n/N* (%)	Leishmania spp.^d^ *n/N* (%)	Rickettsia spp.^e,g^ *n/N* (%)
04/2012-03/2013	6/30 (20)	2/30 (7)	1/30 (3)	1/30 (3)	3/30 (10)	–
04/2013-03/2014	15/47 (31.9)	8/47 (17)	0/47 (0)	6/47 (13)	2/47 (4)	–
04/2014-03/2015	9/67 (13.4)	3/67 (5)	0/67 (0)	6/67 (9)	1/67 (2)	–
04/2015-03/2016	12/58 (20.7)	6/58 (10)	0/58 (0)	2/58 (3)	2/58 (3)	3/45 (7)
04/2016-03/2017	19/87 (21.8)	6/84 (7)	0/84 (0)	3/87 (3)	2/87 (2)	11/87 (13)
04/2017-03/2018	33/99 (33.3)	8/98 (8)	0/98 (0)	10/99 (10)	1/99 (1)	14/99 (14)
04/2018-03/2019	44/98 (44.9)	8/96 (8)	0/96 (0)	22/98 (22)	8/98 (8)	21/98 (21)
04/2019-03/2020	37/138 (26.8)	12/138 (9)	0/138	23/138 (17)	3/138 (2)	3/138 (2)
Total	175/624 (28)	53/618 (9)	1/618 (0.2)	73/624 (12)	22/624 (4)	52/467 (11)

^a^Polymerase chain reaction (PCR), TaqMan® real-time PCR, target: 18S rRNA

^b^PCR, based on Rishniw et al. [13]

^c^Immunoflourescent antibody test (IFAT), MegaFLUO® EHRLICHIA canis (MegaCor Diagnostik GmbH, Hörbranz, Austria; ≥ 1:40 positive)

^d^IFAT, MegaFLUO® LEISH (MegaCor Diagnostik GmbH, Hörbranz, Austria; > 1:64 positive)

^e^IFAT, RICKETTSIA CONORII IFA SLIDE (Viracell, Granada, Spain; > 1:128 positive)

^f^EDTA blood for PCR was not provided for 6/624 cats

^g^Testing for *Rickettsia* spp. was performed from 07/2015 onwards

## Results

### Signalment and stays abroad

Six hundred twenty-four cats were included in this study. Information on the breed was provided for 554/624 cats (89%). There were 20 different breeds of cats, predominantly European shorthairs (423/554 cats, 76%) as well as mixed breeds (71/554 cats, 13%) and Siamese cats (17/554 cats, 3%). The sex of the animal was indicated for 573/624 cats (92%); of these, 308/573 cats (54%) were male and 265/573 (46%) female. The age of the animal was known in 536/624 cases (86%), for whom the median age was 2 years (mean: 3.53 years; range: 0.2–18 years).

Information on time spent abroad was either unavailable or could not be requested retrospectively for 253/624 cats (41%). Eight out of 624 cats (1%) were born in Germany and had never traveled. A travel history was available for 363/624 cats (58%). This included 29 countries, of which Spain (158/363 cats, 44%), Greece (53/363 cats, 15%), and Romania (33/363 cats, 9%) were most frequently named (Table 2). Among this group of cats, 356/363 (98%) were imported to Germany from abroad, of which 38 cats were imported by animal rescue organizations, and 15 cats were imported by private individuals after a holiday. One cat was imported from France and subsequently traveled to Turkey every year with its owner. Six of the 363 cats (2%) were born in Germany and accompanied their owners on vacations abroad, during which they would be allowed to roam freely in the respective foreign country (Spain, *n* = 2; France, Italy, Romania, Bosnia, each *n* = 1).

**Table 2:** Positive test results in 363 cats with known stays abroad and introduction of the “Feline Travel Profile” diagnostic panel from April 2012 until (and including) March 2020 in the LABOKLIN laboratory (Bad Kissingen, Germany)

Country	*N*	N tested positive/*N* total (%)	*Hepatozoon* spp.^c^	*Dirofilaria* spp.^d^	*Ehrlichia* spp.^e^	*Rickettsia* spp.^a, f^	*Leishmania* spp.^g^	Stays abroad
Countries of the European Union								
Spain	158	51/158 (32)	19	–	21	11	5	131 imports, 20 animal welfare imports, 5 imports after holidays, 2 holidays
Greece	52	17/52 (33)	8	–	7	2	2	44 imports, 6 animal welfare imports, 2 imports after holidays
Romania	28	8/28 (29)	1	–	2	5	1	26 imports, 1 animal welfare imports, 1 holiday
Bulgaria	25	7/25 (28)	1	–	5	1	–	18 imports, 6 animal welfare imports, 1 import after holidays
Italy	23	3/23 (13)	–	–	–	3	–	20 imports, 1 import after holidays, 1 animal welfare import, 1 holiday
Croatia	15	3/15 (20)	–	–	2	1	–	11 imports, 4 imports after holidays
Portugal	9	2/9 (22)	1	–	1	–	–	8 imports, 1 animal welfare import
France	4^B^	0/4 (0)	–	–	-	–	–	3 imports^a^. 1 holiday
Cyprus	3	2/3 (67)	1	–	1	–	–	2 imports, 1 animal welfare imports
Malta	2	2/2 (100)	1	–	1	–	–	2 imports
Slovenia	1	0/1 (0)	–	–	–	–	–	1 import
Total EU	320^b^	95/320 (30)	32	–	40	23	8	266 imports, 36 animal welfare imports, 13 imports after holidays, 5 holidays
Total Non-EU	44^a^	15/44 (34)	7	–	–	3	4	39 imports, 2 animal welfare imports, 2 imports after holidays, 1 holiday

^a^*Rickettsia* spp. IFAT was added to the “Feline Travel Profil” from July 2015 onwards

^b^One cat which tested negative was imported from France and subsequently traveled to Turkey every year with its owner

^c^Polymerase chain reaction (PCR), TaqMan® real-time PCR, target: 18S rRNA

^d^PCR, based on Rishniw et al. 2006

^e^Immunoflourescent antibody test (IFAT), MegaFLUO® EHRLICHIA canis (MegaCor Diagnostik GmbH, Hörbranz, Austria; ≥ 1:40 positive)

^f^IFAT, RICKETTSIA CONORII IFA SLIDE (Viracell, Granada, Spain; > 1:128 positive)

^g^IFAT, MegaFLUO® LEISH (MegaCor Diagnostik GmbH, Hörbranz, Austria; › 1:64 positive)

### Laboratory diagnostics

Results from 2951 direct and indirect detection assays on samples from 624 cats were evaluated. PCR testing was performed on samples from 618/624 cats (99.9%) for both *Hepatozoon* spp. and *Dirofilaria* spp. For 6/624 cats (0.1%), no EDTA blood was provided for analysis. Indirect testing *via* IFAT for *Ehrlichia* spp. and *Leishmania* spp. was performed for all 624 cats. After the addition of a *Rickettsia* spp. IFAT to the “Feline Travel Profile” in July 2015, 467/624 cats (75%) were also tested for this pathogen.

One hundred seventy-five out of 624 cats (28%) had positive test results for at least one of the pathogens (Table 1). PCR testing was reported as positive for *Hepatozoon* spp. in 53/618 cats (9%) and for *Dirofilaria* spp. in 1/618 cats (0.2%). IFAT testing showed the following: 73/624 cats (12%) had positive serology for *Ehrlichia* spp., 52/467 cats (11%) for *Rickettsia* spp., and 22/624 cats (4%) for *Leishmania* spp. For *Ehrlichia* spp. serology, titers of 1:40 (*n* = 44), 1:320 (*n* = 24), and 1:640 (*n* = 5) were detected. Antibodies to *Rickettsia* spp. were found in 52 cats, with titers of 1:256 (*n* = 33), 1:512 (*n* = 14) and 1:1024 (*n* = 5). The 22 cats with antibodies to *Leishmania* spp. had titers of 1:128 (*n* = 14), 1:256 (*n* = 3), 1:512 (*n* = 4) and 1:1024 (*n* = 1).

In 22/624 cats (4%), more than one pathogen was found by direct and/or indirect detection methods. This group includes three cats (14%) with positive test results prior to the addition of *Rickettsia* spp. IFAT to the “Feline Travel Profile” (*Leishmania* spp. IFAT/*Dirofilaria* spp. PCR, *Leishmania* spp. IFAT/*Hepatozoon* spp. PCR, and *Leishmania*/*Ehrlichia* spp. IFAT) in July 2015 and 19/22 cats (86%) after this addition. *Rickettsia* spp. were implicated in 14 of these 19 cats (74%). Overall, 19 cats had two concurrent positive test results for different pathogens [*Ehrlichia/Rickettsia* spp. IFAT (*n* = 6); *Leishmania/Rickettsia* spp. IFAT and *Leishmania* spp. IFAT*/Hepatozoon* spp. PCR (*n* = 3, respectively); *Rickettsia* spp. IFAT*/Hepatozoon* spp. PCR, *Ehrlichia* spp. IFAT/*Hepatozoon* spp. PCR, and *Ehrlichia/Leishmania* spp. IFAT (*n* = 2, respectively) as well as *Leishmania* spp. IFAT*/Dirofilaria* spp. PCR (*n* = 1)]. Three cats had simultaneous positive test results for three pathogens [*Ehrlichia/Leishmania/Rickettsia* spp. IFAT (*n* = 2), *Leishmania* spp. IFAT*/Rickettsia* spp. IFAT*/Hepatozoon* spp. PCR (*n* = 1)].

Among the 363 cats with a history of time spent abroad, 110 (30%) had positive test results for at least one vector-borne pathogen. Three hundred twenty of the 363 cats (88%) had been to a different country in the European Union, and 44 (12%) had stayed in countries outside the European Union [primarily Turkey (*n* = 12) and Dubai (*n* = 5)] (Table 2). One cat had been imported from France and subsequently accompanied its owner to Turkey every year, and it was thus included in both categories. Six cats were born in Germany and accompanied their owners on travels abroad, but all had entirely negative test results in this study. Test results were positive for more than two to three pathogens in 10/363 cats (3%), the majority of which had returned or came from Spain (*n* = 5) and Greece (*n* = 2).

There was a negative travel history in 8/624 cats (1%) tested by the “Feline Travel Profile.” Four of these eight cats had antibodies for *Rickettsia* spp.

## Discussion

This study investigated 624 cats in Germany for the presence of *Hepatozoon* spp. and *Dirofilaria* spp. *via* direct detection methods as well as for the presence of antibodies against *Ehrlichia* spp., *Rickettsia* spp., and *Leishmania* spp. *via* indirect detection methods. A background history was available for 371 cats, the majority of which had either been imported or had spent time outside of Germany (363/371 cats, 98%). These numbers can be attributed to the fact that the testing panel used as the basis for this study to detect different vector-borne pathogens is offered as a “Feline Travel Profile” to veterinarians. The majority of the 363 cats with a known background history of time spent abroad had done so in other European countries (88%), but several non-European countries were also implicated (22%, Table 2). Spain (*n* = 158) and Greece (*n* = 52) were most commonly involved, and many of the cats with a background history implicating either one of these countries had positive test results (Spain: 32%, Greece: 33%). Imports by animal welfare organizations may play a significant role for both these countries (Spain: 20 animal welfare imports, Greece 6 animal welfare imports). Similarly, 6 out of 25 cats that had spent time in Bulgaria were imported to Germany by animal welfare organizations (Table 2). The number of imported cats greatly outweighs that of cats accompanying their owners' travels, which contrasts with the findings of previous studies in dogs [14, 15]. The rising numbers of cats tested between 2012 and 2020 (Table 1) may indicate that the import of cats is gaining importance in Germany. Together with the change in climate in many parts of Europe, this could contribute to an increase in the spread of pathogens and their potential vectors into previously non-endemic areas such as Germany, where they may spread further and form reservoirs for infection. Under suitable conditions, pathogens transmitted by imported vectors may cause infection in competent hosts endemic to Germany, of which cats are only one example. Moreover, endemic vectors which are potentially competent may be infected with previously non-endemic pathogens during a bloodmeal on infected cats and could proceed to contribute to the spread of these pathogens. One example of this phenomenon are presently isolated cases of autochthonous infections with *D. repens* [16–18] and *Leishmania infantum* [19] in dogs in Germany.

To the knowledge of the authors, the prevalence of many vector-borne infectious pathogens in cats in Germany is still unknown, as for example for *Hepatozoon* spp. In this study, 9% of the cats tested for this pathogen had positive PCR results. Direct detection methods demonstrate the presence of deoxyribonucleic acid or the antigen of a pathogen. Apart from infections with *H. canis* and *H. silvestris*, *H. felis* seems to be the primary infecting pathogen in cats [20–25]. Species differentiation showed the presence of *H. felis* in 7/53 cats infected with *Hepatozoon* spp. in this study. They had been imported from Spain (*n* = 5) and from Greece and Malta (*n* = 1 respectively), which is consistent with the above-cited literature. There is little knowledge about the pathogenesis, replication cycle, host range, and modes of transmission of *Hepatozoon* spp in cats. In addition to vector transmission, there are reports of transplacental transmission of *H. canis* and *H. felis* [6]. Therefore, any female cat that tested positive in this study and was not spayed (*n* = 7) could transmit the pathogen to its kittens in Germany, whether or not there was any contact with a vector. Autochthonous infection with *H. felis* has been reported in a cat in Austria [9]. This may indicate the spread of the pathogen and/or vectors from historically endemic countries in the Mediterranean to more northern regions of Central Europe. In this study, 39/53 cats with positive test results had a history of travel/import to a known endemic area, and time spent abroad could not be excluded for any of the animals with positive test results. Consequently, this study provides no evidence of autochthonous infections in cats within Germany.

One cat in this study had positive PCR results for *Dirofilaria* spp., but further species differentiation was not done, and a travel history or information on any time spent abroad was not available. This cat also had a positive IFAT for *Leishmania* spp., so contact with the pathogens in an endemic country in the Mediterranean is likely. Infections with *Dirofilaria* spp. in cats and dogs historically occur in Mediterranean countries but have recently spread within these countries, such as for example Italy, Spain, France, Greece, and Turkey [10]. *Dirofilaria repens* [26–28] has been the primary pathogen reported in Central and Eastern Europe, and it is currently considered an emerging zoonotic agent in all of Europe [29]. The prevalence of *Dirofilaria* spp. in cats varies from 0 to 33% across Europe [11, 30–40]. According to predictive models developed for dirofilariasis, temperatures during the summer may be suitable for the life-cycle of larvae in mosquitoes even in colder regions like the UK, provided that reservoirs are present [10, 27, 41, 42]. The true prevalence of *D. immitis* may be higher than indicated by the relatively low number of cats with positive test results in this study. Many of the immature pathogens are destroyed shortly after reaching the pulmonary arteries in cats, and the lifespan of the surviving pathogens is shorter in cats (2–4 years) than in most other species, such as dogs (5–7 years) [43]. Cats are rarely infected with more than five roundworms, which can be missed even in a post-mortem examination [44]. Microfilaremia is rare in cats, as fewer male worms are present [44]. Data on the prevalence of *Dirofilaria* spp. in cats in Germany are not yet available. A single case report from Central Europe describes a cat in Poland which was infected with *D. repens* and *Wolbachia* spp. [24].

Indirect detection methods were used to detect *Ehrlichia* spp., *Rickettsia* spp., and *Leishmania* spp. They only demonstrate the presence of antibodies produced in response to the pathogen contact, but not necessarily the presence of disease. It is generally possible to distinguish more recent infections from those in the past by means of simultaneous immunoglobulin M levels or paired serum samples taken at intervals of 2 to 4 weeks [45]. The indirect IFAT utilised in this study detected immunoglobulin G antibodies for all pathogens. Furthermore, the interpretation of IFAT can be subjective, so the sensitivity can be low, especially when titers are low or borderline. Limitations may also include the possibility of cross reactivity with other pathogens (e.g. between all *Ehrlichia* species [45]) as well as false-negative results in very young or immunosuppressed animals or where investigations were done early in the natural history of the disease and therefore prior to seroconversion [46]. Considering all limitations of antibody assays, positive results can be correlated with given antigen exposure.

The IFAT used in this study detected antibodies to *Leishmania* spp. in 22/624 cats (4%). Cats in Mediterranean countries are generally infected by *L. infantum*. There is much variation in the reported prevalence of *Leishmania* spp. in cats tested by indirect assays not only between different European countries but also across different regions within one country, ranging from 0.1% to 60% [1, 30, 40, 47–69]. Currently, dogs are the only known primary reservoir of *Leishmania* spp. [70]. Sandflies can be infected with *L. infantum* during a bloodmeal on an infected cat. Therefore, cats may be instrumental in the spread of the pathogen in areas with a high prevalence [71], even though their role regarding the transmission cycle of the pathogen is still unknown [72]. The presence of competent vectors such as *Phlebotomus perniciosus* has been reported in the south of Germany [72], as has the potentially competent vector *P. mascitti* [73, 74]. There is little evidence on the susceptibility or resistance of cats to natural infection. Cats have a more efficient T-helper 1 cell immune response compared to dogs, which may be the cause of the lower prevalence of the pathogen in cats [47]. Twelve out of the 22 cats with positive IFAT results (55%) in this study were imported to Germany from Mediterranean countries and Southeast Europe, where *L. infantum* is endemic. One out of the 22 cats (5%) was imported from Brazil, where cats may be infected by not only *L. infantum* but also *L. amazonensis* or *L. braziliensis* [75–78].

Antibodies against *Ehrlichia* spp. were detected *via* IFAT in 12% of the tested cats. Previous studies involving indirect detection methods (IFAT) report a 1–18% prevalence of *Ehrlichia* spp. in cats in the Mediterranean area [30, 51, 53, 55, 58, 79–83]. Data on the prevalence of antibodies against *Ehrlichia* spp. in cats in Germany are not currently available. IFAT may show some cross-reactivity with *E. chaffensis* (found in cats in the US and Brazil) and *E. ewingii* (found in cats in the US) as well as with *A. phagocytophilum* and *A. platys* at lower titers. Cross-reactivity due to contact with *A. phagocytophilum* in Germany cannot be excluded, especially in the 44 cats with a low titer of 1:40 in this study.

Eleven percent of 467 cats had positive IFAT results for *Rickettsia* spp. Seroprevalence in cats has been reported in Italy, Spain, and Portugal (IFAT/ELISA: 0-48.7%) [30, 47, 51, 53, 79, 84, 85]. Cats may be instrumental in the transmission cycle of some rickettsiae of the spotted fever group (SFG), especially *R. conorii* and *R. felis* [86, 87]. Antibodies for *R. conorii* have been detected in cats after infestation with *Rhipicephalus sanguineus* [79, 84, 87], making vector contact in the Mediterranean most likely. Regarding *R. felis*, the situation differs: cats will have antibodies after being infected (either naturally or experimentally) with fleas of the species *Ctenocephalides felis* [88]. The pathogen has also been detected *via* PCR in previously non-infected fleas after a bloodmeal on infected cats [89]. Consequently, *C. felis* can be considered a competent vector, and autochthonous infections within Germany are possible. This study detected antibodies by means of IFAT, which is regarded as the gold standard for serological confirmation of pathogen contact in dogs and cats. There are, however, cross-reactions between any of the more than 20 species in the spotted fever group [87]. We detected antibodies to *Rickettsia* spp. in 52/467 cats (11%). Twenty-nine cats were seropositive and had been imported from abroad, and it is unclear whether they were infected in Germany or in their country of origin. The four seropositive cats which had never left Germany were most likely infected with *R. felis.* The clinical importance of *Rickettsia* spp. infections in cats is still unknown. A study in clinically symptomatic cats found no association between positive antibody titers and fever, and no febrile cats in this study had positive PCR results for *R. felis* or *R. rickettsi* [90].

In this study, 22 out of 624 cats (4%) had positive test results for more than one pathogen. It is known that co-infections may complicate diagnosis and treatment in dogs and may worsen their prognosis [2]. Coinfections with multiple vector-borne pathogens may occur in cats as well as dogs and humans, but their clinical consequences are still unknown and should be evaluated in further studies, especially in cats [45]. Nine cats infected with *Hepatozoon* spp. also had antibodies against *Leishmania* spp. (*n* = 4), *Rickettsia* spp. (*n* = 3), and *Ehrlichia* spp. (*n* = 2). Antibodies to *Leishmania* and *Ehrlichia* spp. were present in 12 cats infected with *Hepatozoon* spp., respectively. This indicates a pathogen contact with concurrent immunosuppression to be discussed in case of persistent infection, as it may result in increased susceptibility of infected animals to other pathogens [2].

## Limitations of this study

Limitations of this study are mainly its retrospective design (e.g. no consistent histories) and the limited number of pathogens included. Certain vector-borne infectious pathogens such as *Cytauxzoon* spp. could not be included. Furthermore, species differentiation for specific pathogens included in the study was not performed, except in the case of seven cats with positive test results for *H. felis*. There was also no information on the extent of ectoparasite prophylaxis in the cats, which may impact the prevalence of certain vector-borne pathogens. In the cats which had traveled with their owners to endemic countries, it was not possible to reliably document the duration or the time of the year of these travels. As many of the relevant vectors show pronounced seasonality, the time of year may significantly influence both incidence and prevalence of the pathogens they may transmit. The histories taken from the veterinarians only included the countries of stays abroad.

## Conclusions

Of the cats included in this study, 28% had positive test results for at least one vector-borne pathogen. As vector-borne infections often remain undiagnosed, it is important to take thorough histories of any time spent abroad in all cats in which vector-transmitted infections are suspected. Owners of imported cats, or those who choose to take their cats with them on holidays abroad, should be given detailed information on any and all potential infections and resulting risks. Ectoparasite prophylaxis is advisable in all cats. The zoonotic potential of some pathogens such as *L. infantum*, *D. immitis*, and *D. repens* and their resulting importance in human medicine has to be noted [2].

## Data Availability

All data generated or analyzed during this study are included in this published article. Parts of this study were presented as a poster at the DVG Congress for Internal Medicine and Laboratory Diagnostics in Gießen, Germany (30 January–01 February 2020) and as an oral presentation at the International Research Conference on Veterinary Parasitology and Entomology in Copenhagen, Denmark (11–12 June 2020, Online Congress).

## Abbreviations

DAT: Direct agglutination test
DNA: Deoxyribonucleic acid
EDTA: Ethylenediaminetetraacetic acid
FeLV: Feline leukemia virus
FIV: Feline immunodeficiency virus
IFAT: Indirect immunofluorescence test
PCR: Polymerase chain reaction
